# What it takes to save lives: An assessment of water, sanitation, and hygiene facilities in temporary COVID-19 isolation and treatment centers of Southern Ethiopia: A mixed-methods evaluation

**DOI:** 10.1371/journal.pone.0256086

**Published:** 2021-08-13

**Authors:** Aiggan Tamene

**Affiliations:** Environmental Health Unit, School of Public Health, College of Medicine and Health Sciences, Wachemo University, Hossaena, Ethiopia; San Giuseppe Hospital, ITALY

## Abstract

**Background:**

Quality water, sanitation, and hygiene facilities act as barricades to the transmission of COVID-19 in health care facilities. These facilities ought to also be available, accessible, and functional in temporary treatment centers. Despite numerous studies on health care facilities, however, there is limited information on the status of WASH facilities in such centers.

**Methods:**

The assessment of health care facilities for the COVID-19 response checklist and key informant interviews, were used for data collection. 35 treatment centers in Southern Ethiopia were surveyed. Eightkey informants were interviewed to gain an understanding of the WASH conditions in the treatment centers. The Quantitative data was entered using EPI-INFO 7 and exported to SPSS 20 for analysis. Results are presented using descriptive statistics. Open Code 4.02 was used for the thematic analysis of the qualitative data.

**Results:**

Daily water supply interruptions occurred at 27 (77.1%) of the surveyed sites. Only 30 (85.72%) had bathrooms that were segregated for personnel and patients, and only 3 (3.57%) had toilets that were handicapped accessible. 20(57.2%) of the treatment centers did not have a hand hygiene protocol that satisfied WHO guidelines. In terms of infection prevention and control, 16 (45.71%) of the facilities lacked adequate personal protective equipment stocks. Between urban and rural areas, there was also a significant difference in latrine maintenance, hand hygiene protocol design and implementation, and incineration capacity.

**Conclusion:**

The results reveal crucial deficiencies in the provision of WASH in the temporary COVID-19 treatment centers. Efforts to improve WASH should offer priority to hygiene service interventions to minimize the risk of healthcare-acquired infections. The sustainable provision of hygiene services, such as hand washing soap, should also be given priority.

## Introduction

Coronavirus Disease 2019 (COVID-19), an infectious disease caused by severe acute respiratory syndrome Coronavirus 2 (SARS-CoV-2), first appeared in China in December 2019. The virus spreads mainly through respiratory droplets between people when an infected person coughs, sneezes, or touches surfaces or objects [1, 2]. Information on the transmission of the COVID-19 virus is ever-evolving, and the severity of infection with the virus has been shown to vary from very mild, non-respiratory symptoms to severe acute respiratory diseases, resulting in organ failure, sepsis, and death [3]. The majority of people (80%) recover from the disease without requiring special treatment. It can, however, lead to severe illness for certain individuals. Approximately 1 in 5 people who are infected with COVID-19 have trouble breathing and need hospital treatment [2, 4, 5].

The danger of contracting COVID-19 is now higher than it has been since the onset of the pandemic. People have become complacent as they have gotten weary of preventive measures, and this complacency is reflected in infection and death rates [6]. The pandemic and accompanying global crisis have had direct and indirect repercussions on Sub-Saharan Africa (SSA), particularly Ethiopia [7].

Ethiopia is only second to South Africa in terms of recorded cases and deaths in Sub-Saharan Africa, with an overall case fatality ratio (CFR) of roughly 1.5% compared to around 2.2% in the rest of the globe [8]. The lower case-fatality ratio (CFR) is attributed in part to the Ethiopian population’s younger age structure, with only 5% of people over 60 years old (compared to over 20% in most of Europe). However, high infection rates in the old, which result from extensive intergenerational mixing and a larger number of contacts found among the elderly, partially offset the benefit of a younger population [9].

With 116 million people, the country is Africa’s second-most populous nation. The first case of COVID-19 was recorded on March 13, 2020. Since then, cases have increased slowly but steadily. It took 79 days to reach the first 1000 cases, but in the next 9 days, that number doubled [8]. As of June 9th, 2020, the country recorded 273,145 cases and conducted 2.7 million lab tests, accounting for around 2.3% of the total population. This suggests that the number of confirmed cases may not be an accurate reflection of reality, particularly when estimating the amount of community transmission.

The WHO’s initial assessment of preparedness in Ethiopia found numerous flaws in COVID-19 intensive care capabilities [8]. Even before the pandemic, Ethiopia’s poverty level was extremely high. In 2019, 81.3% percent of Ethiopia’s population was living in multidimensional poverty [7]. We can deduce from this that, depending on the pandemic’s trajectory, counter-measure outcomes, and underlying and structural issues, the socio-economic consequences already being felt across Ethiopia have the potential to increase.

At present, there is a three-tiered system of COVID-19 centers in Ethiopia. Tier one is for people with COVID-19 but unable to stay at home. This entails minimal supervision and primary self-care. Tier two is a low-acuity site for individuals needing some supervision and additional treatment. Tier 3 is home to high-acuity patients who need comprehensive surveillance and respiratory treatment with highly skilled care staff [10]. Although the conversion of existing buildings into fully functional hospitals is not practical, public and private hotels, colleges, and universities have been converted to first and second-tier treatment centers within the country. These temporary patient care facilities relieve the burden on mainline hospitals by providing more space for patient beds [8, 11, 12].

In our current COVID-19 climate, speed and efficiency are key design drivers—but the safety of patients and staff is also of paramount importance. Clean water, sanitation, and hygiene (WASH) programs are one of the ways to ensure such safety, and an investment in water and sanitation systems, is one of the most cost-effective pandemic preparedness strategies, especially in resource-constrained settings [13]. In health care facilities (HCFs), WASH is characterized as infrastructure, services, and behaviors that encompass water supply and water quality, sanitation facilities (including bathing or shower areas), soap and hand wash water availability, and certain elements of waste management in health care (e.g. waste bins, waste treatment facilities) [14].

In a 2019 global report of WASH in HCFs, one in four HCFs lacked basic water services, one in five did not have sanitation services, and one in six had no hygiene services [15]. In the local context, water coverage in such facilities is 32% and the sanitation coverage is 85% while the hygiene data are not available [13]. Quality WASH facilities act as barricades to the transmission of the COVID-19 virus in health care facilities [16]. These facilities ought to also be available, accessible, and functional in temporary HCFs and quarantine centers [17].

With around 22 million people, Ethiopia’s Southern Nations, Nationalities, and Peoples’ Region (SNNPR) accounts for 20% of the country’s population. There are 18 administrative zones and 7 special districts (an administrative subdivision that is equivalent to an autonomous area and is not part of a zone) in the regional state. Although the region’s age distribution is comparable to national figures, the SNNPR is Ethiopia’s most rural region, with poverty rates four times greater than in urban areas [18].

The region has 79 hospitals, 729 health centers, and 3961 health posts for the delivery of health services. In addition to the traditional health infrastructure, there are 35 temporary COVID-19 isolation and treatment centers, as well as two test labs. Despite numerous studies on infection control and hygiene practice in health care facilities, however, there is limited information on the status of WASH facilities in temporary centers. It is against this background that, this article aims to examine the availability, accessibility, functionality, and disparity of water, sanitation, and hygiene (WASH) facilities in temporary COVID-19 isolation and treatment centers of southern Ethiopia in 2020.

## Methods and materials

### Study area and design

A facility-based mixed design study was conducted in the Southern Nations, Nationalities, and Peoples’ Region (SNNPR), from October 25 to December 22, 2020.

### Sample size determination

There are a total of 35 temporary COVID-19 isolation and treatment centers in southern Ethiopia. According to information obtained from the regional health bureau, 21 temporary facilities were converted from government-owned institutions to treatment centers and 14 were private facilities converted to temporary treatment centers. After obtaining the list and location of these temporary sites, field visits were conducted. Field visit data showed that a total of 2197 people were seeking care in the centers and that 368 health workers were employed in the temporary facilities, 38 of whom were infection prevention and patient safety officers whose roles were most closely linked to WASH operations.

### Sampling procedure

All 35 temporary treatment centers were included in the current analysis for the quantitative study, as it was noted that data quality, viability, and resources available would not be affected. For the key informant interviews, six hospitals were purposely chosen as a consequence of their large COVID-19 patient flow. Three government-owned buildings converted into treatment centers, and three former private-owned buildings now operating as treatment centers were included. Eight key-informant interviews were conducted. Six, with infection prevention and patient safety officers at the selected sites and 2, with relevant stakeholders at the Federal Ministry of Health COVID-19 Taskforce and SNNPR COVID-19 Taskforce.

### Data collection tool and procedures

In addition to the main researcher, the research team consisted of three environmental health and safety data collectors and one trained field supervisor. An adopted observational checklist was used to collect data on water, sanitation, and hygiene facilities in the treatment center [19]. For the qualitative data, semi-structured key informant interviews were used to gather data in this research. After a detailed review of the related literature, the research team developed the guides used in this study (S1 File). The interviewees for this study were chosen using an updated, purposive sampling method. The researcher, who chose infection prevention and patient safety officers at random, was provided a list of all infection prevention and patient safety officers at the treatment centers that were chosen.

The goal of the study was explained to the participants before they signed informed consent forms. The interviews lasted between 25–30 minutes; probing questions were used when the responses were vague or ambiguous, or to obtain more specific or in-depth information. The interviews were performed until the data reached saturation, a stage where recurring patterns were evident in the participants’ narratives. Until saturation was achieved, a total of 8 interviews were carried out. The interview consisted of an interviewer, a note-taker, and an observer, in addition to the study participants. During the data collection, face masks and physical distancing protocols were observed.

### Operational definitions

**Latrine availability**: for inpatient settings, one per 20 users (per ward); at least four toilets per outpatient setting (one for staff and one for patients: one for women, one for men, and one for children) [20].

**Latrine accessibility**: latrine should be on the grounds of the facility, toilets should be accessible on all floors in the multi-story building, and routes used to enter toilets should be straight and level for easy access for people in wheelchairs [21].

**Latrine Functionality**: latrine providing facilities with a usable lockable door for the user for privacy; toilet should be accessible inside the facility ground and not limited to the use of staff only [20].

**Hand washing facility availability**: to encourage them to use water as often as required, water points should be sufficiently close to users. Alternatively, a handwashing basin, soap, and a clean water jug can be placed on a trolley used for rounds to promote handwashing between patient contacts as often as necessary [21].

**Hand washing facility functionality**: handwashing basin that can be operated by hand or elbow and has drainage line connected to the sewer system [21].

**Water facility accessibility**: in the healthcare environment, sufficient water collection points and water-use facilities are available to allow easy access to and use of water for medical activities, drinking, personal hygiene, food preparation, laundry, and cleaning [20].

**Improved water supply**: there is an improved water source or water supply throughout the year in all treatment wards and waiting areas for drinking, personal hygiene, medical activities, cleaning, laundry, and cooking purposes [21].

**Improved sanitation facilities**: there are latrines within the facility that can separate human excreta hygienically from human contact while being functional [21].

**Improved hygiene facilities**: availability of hand wash basin with running water and soap (or hand rubs based on alcohol) in all the main areas of the facility to ensure safe hand hygiene [20].

### Data quality control

First, to improve measurement precision, the checklist was prepared in English and translated into the local (Amharic) language, the approach in the translations stressed cross-cultural and conceptual translations rather than literal or linguistic equivalence of the terminologies. Afterward, an English language expert conducted a back-to-back translation into English to verify the accuracy. Also, one-day training on the study purpose and methods of data collection was given for both data collectors and the supervisor. The data collection tool was then pre-tested on 2 temporary isolation and treatment centers outside of the study area in the neighboring Sidama region. The main purpose of the pre test was to identify any problems regarding the design and readability of the tool. A secondary objective of the pretest was to ensure that the instruments were interpretable by individuals with or without WASH knowledge. Interview locations (private instead of at the workplace) and question order modifications were done after the pretest and before components of the tools were finalized, produced and disseminated. The data from the pretest was not included in the main study.

### Data management and analysis

#### Quantitative data analyses

EPI INFO 7 was used to enter the data, which was then exported to SPSS 21 for analysis. Descriptive statistics with frequencies are used to present the study results. Furthermore, comparisons between facilities were done after establishing crucial baseline variables that can aid in facility classification and grouping. Because rural Ethiopia has the lowest access to clean water sources in Sub-Saharan Africa and 59% of rural settlements utilize unimproved toilet facilities [22], an urban/rural classification was deemed the most appropriate. As a result, Chi-square tests were used to check if any significant disparities in WASH facilities existed between urban and rural areas.

#### Qualitative data analyses

Open code 4.03 was used for qualitative data analysis, which followed a thematic framework. The gathered information was transcribed verbatim in the local language (i.e., Amharic). It was later translated by the same person into English. The original transcript was analyzed with its translated edition, following the translation. This meant that the translated items lacked contradictions. Next, data were validated and themes were developed based on the issues arising from the interviews in an inductive and deductive method. The emergent themes were categorized under the subsections of the observational checklist (water quality, sanitation, hygiene, biomedical waste management, and environmental hygiene). To assist in the interpretation of the data when presenting the data, related verbatim quotes are used.

### Ethics approval and consent to participate

Ethical clearance was received from the Ethical Review Committee at Wachemo University. The requisite support letter was obtained from the Health Bureau of SNNPR. Permission from all treatment centers was obtained before the study. Written consent was given by all participants. All participants were advised of the intent of the study and were notified of their right to not participate in the study or to stop the interview.

## Results

The study included all 35 temporary COVID-19 isolation and quarantine centers in the SNNPR. Throughout the region, these treatment centers are scattered and help ease the burden on the state’s regional and federal hospitals. A majority, that is, 21 (60%) of the treatment centers were government-owned buildings converted to treatment centers. Temporary treatment centers received an average of 200 patients per week at present. Concerning their location, 9 (25.7%) of the treatment centers were in rural areas of the region while the rest were located in an urban setting. During the data collection period, 88% of the treatment centers had at least one environmental health officer; while the others were forced to transfer some of their WASH-related duties to the other professionals in the centers (see Table 1).

**Table 1:** General information on temporary COVID-19 treatment centers, in Southern Ethiopia, 2020.

Categories for variables	Frequency (n = 35)	Percentage
**Prior ownership of treatment center**		
Government-owned building	21	60%
Private-owned building	14	40%
**COVID-19 patient intake capacity**		
> 200 beds	10	28.5%
< 200 beds	25	71.5%
**Average weekly intake**		
> 50 cases	11	31.4%
< 50 cases	24	69.6%
**Environmental health officer**		
Yes	31	88%
No	4	12%
**Number of health care workers**		
> 20 HCWs	16	45.7%
< 20 HCWs	19	54.3%
**Treatment center location**		
Urban	16	45.7%
Rural	19	54.3%

*‘‘There are no environmental health professionals for some of the treatment centers’ WASH programs*. *It is conducted by nurses*. *The nurses took 5 days of training on infection prevention protocols and took on the responsibilities as it was hard for us to find people willing to fill the vacancies*.”

*(Regional WASH-Taskforce officer)*

### Availability, accessibility, and functionality of water supply in temporary treatment centers

Even with some discontinuity, all 35 of the surveyed temporary treatment centers had water piped into their facilities. Of the 35 temporary treatment centers, an overwhelming majority, i.e., 27 (77.1%) had daily water supply interruptions (Table 2). One respondent, for instance, expressed dissatisfaction with the lack of critical water supply to the treatment facility, as mentioned below.

**Table 2:** Availability, accessibility and, functionality of water supply in temporary treatment centers of Southern Ethiopia, 2020.

Categories for variables	Frequency (n = 35)	Percentage
**Insufficient water quantity for all the daily needs in the health facility**		
YES	20	57.1%
NO	15	42.9%
**Daily interruptions in the water supply at the health facility**		
YES	27	77.15%
NO	8	22.85%
**Insufficient water storage (less than 24 hours of backup supply)**		
YES	19	54.28%
NO	16	45.72%
**Water is from an unimproved source or sources of contamination (latrines, waste, pollution, etc.) within 10m / 33ft of the water source**		
YES	7	20%
NO	28	80%
**Water is un-chlorinated, insufficiently chlorinated (no chlorine smell or taste in the water at the tap), or is turbid (cloudy)**		
YES	11	31.42%
NO	24	68.58%
**Broken water pipes or uncovered or unsanitary water reservoirs**		
YES	6	17.14%
NO	29	82.86%
**Drinking water for staff, visitors, and patients is not safe and/or in inadequate quantity**		
YES	27	77.15%
NO	8	22.85%

*‘‘There is a frequent discontinuity of water but*, *there is a backup tanker for our services*. *The water and sewerage service authority should focus on the continuous water supply to the treatment center*.*”*

*(Infection prevention and control officer* #*1)*

Another added,

‘‘*We mostly use off-site water sources*. *Such water source limits the amount of water available to the HCF because it must be transported*. *It raises the danger of contamination since water must be stored and transported*, *and adds time to the HCF because people must travel to the water source to collect and transport water*.*”*

*(Infection prevention and control officer* #*1)*

Seven (20%) of the treatment centers used water from an unimproved source. This figure was comparable to the number of treatment centers whose water sources were within 10metersof contaminants (latrines, waste, pollution, etc.). Also, un-chlorinated or insufficiently chlorinated water was used by 11 (31.42%) treatment centers.

*‘‘We use water from a borehole as a replacement when there is discontinuity of line water*. *This makes the water we use less clean than conventional tap water but we deal with what we have*.*”*

*(Infection prevention and control officer* #*2)*

### Availability, accessibility, and functionality of excreta disposal system

All 35 of the temporary treatment centers had bathrooms within their premises. However, only 30 (85.72%) of the centers had toilets separated for staff, patients, and visitors.

*‘‘Before the pandemic*, *these temporary treatment centers were everyday buildings designed for everyday situations*. *When converting such buildings into a health care environment we did come across major problems regarding the adequacy of latrines*.*”*

*(Regional COVID-19 taskforce officer)*

Similarly, although, 88.57% of the assessed toilets had functional doors, and dust bins only three (3.57%) of the treatment centers had toilets that were accessible for people with disabilities.

*‘‘This is a University building*; *it has latrines built to accommodate the disabled but it has been in use for some 50 plus years*. *It is dilapidated*.*”*

*(Infection control and prevention officer* #*4)*

*‘‘Hotels provide possibly the most feasible places for temporary healthcare facilities because they provide individual rooms with personal restrooms*, *which can help stop cross-contamination*. *College dorms have the comparable infrastructure already in place as well*. *However*, *convention centers and similar high-occupancy venues have beenhard to set up because of the inadequate availability of*, *amongst other things*, *latrine facilities*.*”*

*(Federal COVID-19 taskforce officer)*

Regarding the availability of dedicated handwashing stations with water and soap or chlorinated water, 12 (34.97%) of the treatment centers had no running water for patient latrines due to the unavailability of piped water (Table 3).

**Table 3:** Availability, accessibility, and functionality of excreta disposal system in temporary treatment centers of Southern Ethiopia, 2020.

Categories for variables	Frequency (n = 35)	Percentage
**The facility does not have toilets separated for staff, patients, and visitors**		
YES	5	14.28%
NO	30	85.72%
**Some units do not have dedicated latrines**		
YES	35	100%
NO	0	0%
**Existing toilets are not separated by sex and for people with reduced mobility**		
YES	32	94.3%
NO	3	3.57%
**Latrines are not maintained properly**		
YES	18	(51.4%)
NO	17	(48.6%)
**Latrines are not regularly (every 2–3 hrs) disinfected**		
YES	25	71.42%
NO	10	28.58%
**Not all latrines have dedicated handwashing stations with water and soap or chlorinated water**		
YES	12	34.97%
NO	23	65.73%
**Some pits and/or septic tanks are full**		
YES	6	17/14%
NO	29	82.86%

*‘‘Most HCFs store their water in a covered storage container*, *the majority of latrine users fail to safely collect water from these containers*. *Instead*, *they use a cup*, *bowl*, *or their hands to scoop water from the container*, *which might introduce contaminants*.*”*

*(Regional COVID-19 taskforce officer)*

### Availability, accessibility, and functionality of a shower and laundry system

During a pandemic marked by the need for cleanliness, it makes sense that the shower and laundry system of any treatment center is of the utmost importance. In the present study, at the time of data collection, almost all (94.7%) of the laundries in the treatment facilities were not functional. This subjected the laundry employees to the manual washing of each piece of linen that found its way into their department.

*‘‘Nowadays*, *our workers toil hard to make up for the downed washing machines this manual washing puts them and their families at great risk*.*”*

*(Infection control and prevention officer* #*3)*

Similarly, 29 (82.85%) of the treatment centers had showers that were not functional and/or not maintained properly (with 0.5% of chlorine after each patient) (Table 4).

**Table 4:** Availability, accessibility, and functionality of a shower and laundry system in temporary treatment centers of Southern Ethiopia, 2020.

Categories for variables	Frequency (n = 35)	Percentage
**Some units don’t have dedicated showers**		
Yes	20	57.1%
No	15	42.9%
**Showers are not functional or maintained properly(with 0.5% chlorine after every use)**		
Yes	29	82.25%
No	6	17.14%
**The laundry area is not functional**		
Yes	33	94.28%
No	2	5.72%
**Some soak-away pits are full**		
Yes	22	62.85%
No	13	37.15%

### Availability, accessibility, and functionality of handwashing stations

In the current study, 26 (74.28%) of the treatment centers had functional handwashing points in service areas and in any location where healthcare is delivered. Soap stands were also available at each hand washing facility, for the majority i.e., 28(80%) of the treatment centers. Similarly, while 21(60%) of the handwashing stations had running water, the rest stored water near hand washing facilities (Table 5).

**Table 5:** Availability, accessibility, and functionality of handwashing stations in temporary treatment centers of Southern Ethiopia, 2020.

Categories for variables	Frequency (n = 35)	Percentage
**Absence of soap or chlorinated water at any hand washing locations**		
YES	7	20%
NO	28	80%
**Absence of posters reminding users of correct handwashing procedures**		
YES	14	40%
NO	21	60%
**Absence of functional handwashing points in any location where healthcare is delivered or service areas**		
YES	9	25.72%
NO	26	74.28%
**Absence of hygiene promotion and hand washing supplies and stock-piling**		
YES	18	51.42%
NO	17	48.58%
**Hand hygiene protocol is properly designed and applied regularly**		
YES	15	42.8%
NO	20	57.2%

For facilities that did not have such services, one key informant explained that more pressing administrative needs such as the procurement of protective equipment were given priority given the costs that go into repairing broken facilities.

*‘‘Three of the sinks are broken and we closed access to them*. *We can’t afford to fix the broken stations every single time; we have more urgent requirements like protective equipment*.*”*

*(Infection control and prevention officer* #*3)*

Another added,

*‘‘We put soap on every handwashing sink every day*, *but the soap is taken by the patients within a few minutes; we can’t replace it every minute*. *Not in this poor district*.*”*

*(Infection control and prevention officer* #*6)*

### Availability, accessibility, and functionality of a waste disposal system

A small number of the treatment centers surveyed 6(17.14%) had insufficient, and/or overflowing containers for waste disposal. Likewise, 7(20%) of the evaluated centers had no mechanism for hazardous waste separation. The shortage of waste disposal containers was linked by one infection prevention and patient safety officer to the former purposes the buildings served.

*‘‘They (the owners of private buildings) concentrate only on their business instead of WASH facilities*. *On the grounds of this house*, *there is one waste collection bin*. *The general waste is collected once a week… The container gets full and overflows in between pickups*.*”*

*(Infection prevention and patient safety officer* #*2)*

The temporary treatment centers were also assessed for their incinerator capacity with 21(60%) failing to have sufficient incineration capacity compared to the waste generated (Table 6).

**Table 6:** Availability, accessibility, and functionality of a waste disposal system, in temporary treatment centers of Southern Ethiopia, 2020.

Categories for variables	Frequency (n = 35)	Percentage
**Insufficient, inadequate, or overflowing waste disposal containers**		
YES	6	17.14%
NO	29	82.85%
**No sources of separation of hazardous wastes**		
YES	7	20%
NO	28	80%
**Medical wastes observed in health facility grounds or public spaces or medical waste disposal area unfenced**		
YES	2	5.72%
NO	33	94.28%
**Incinerator capacity is not sufficient compared to the waste generated by the HCF**		
YES	20	57.2%
NO	15	42.8%
**Pools of standing water were observed at water points**.		
YES	23	65.71%
NO	12	34.29%

*‘‘There are lots of design gaps in this treatment center*. *However*, *since the building is registered as a cultural and tourism site; it is difficult to get a construction permit to build a more compatible incinerator*.*”*

*(Infection prevention and patient safety officer* #*4)*

### Availability, accessibility, and functionality of an infection prevention and control system

Concerning the infection prevention and control scheme in place, 16 (45.71%) of the treatment facilities assessed were inadequately supplied with personal protective equipment (gloves, overalls, masks, etc.).

*‘‘We survive with our existing gear because the pandemic has strained all available resources*. *We take what we can get and use it to our full advantage; the government provides what it can*.*”*

*(Federal COVID-19 taskforce officer)*

7 (20%) of the temporary quarantine and isolation centers had a shortage of cleaning equipment (buckets, mops, etc.) and/or disinfectant solutions, while 8 (22.85%) lacked hand disinfectants (with hand sanitizer or 0.5% chlorine solution in the ward and 0.05% outside) (Table 7).

**Table 7:** Availability, accessibility, and functionality of an infection prevention and control system, in temporary treatment centers of Southern Ethiopia, 2020.

Categories for variables	Frequency (n = 35)	Percentage
**Daily disinfection of beds, floors, walls, equipment, surfaces**		
YES	9	25.71%
NO	26	74.29%
**Disinfection stations (with hand sanitizer or 0.5% chlorine solution in the ward and 0.05% outside)**		
YES	27	22.85%
NO	8	77.15%
**Cleaning equipment (buckets, mops, etc.) or disinfectant solutions**		
YES	28	20%
NO	7	80%
**Personal protection equipment (gloves, overalls, masks, etc.) for staff**		
YES	16	45.71%
NO	19	54.29%
**Lack of or insufficient stock of IPC-related supplies**		
YES	16	45.71%
NO	19	54.29%

### Disparities in WASH services among urban and rural isolation and treatment centers

The Chi-square test was used to see if there was a significant difference in WASH services between rural and urban isolation and treatment centers. Per the findings, there is a considerable disparity in latrine maintenance, hand hygiene protocol design and implementation, and incineration capacity (Table 8).

**Table 8:** Chi-square analysis for the study of disparities of water, hygiene, and sanitation status among rural and urban facilities in southern Ethiopia, 2021.

Variables	Location			Test Statistic	
	Rural n (%)	Urban n (%)	Total n (%)	χ2	Sig(p)
**Hand hygiene protocol is properly designed**				**4.644**	**0.035**
Yes	5 (26.3%)	10 (62.5%)	15 (42.8%)		
No	14 (73.4%)	6 (37.5%)	20 (57.2%)		
**Latrines are not maintained properly**				**6.556**	**0.031**
Yes	13 (68.4%)	5 (31.2%)	18 (51.4%)		
No	6 (31.6%)	11 (68.8%)	17 (48.6%)		
**Incinerator capacity is not sufficient**				**4.638**	**0.030**
Yes	14 (73.4%)	6 (62.5%)	20 (57.2%)		
No	5 (26.3%)	10(37.5%)	15 (42.8%)		

In the present study, there was a significant difference in the failure to consistently maintain latrines between urban and rural isolation and treatment centers (urban = 5 (31.2%), rural = 13 (68.4%), **χ2** = 6.556, sig (p) 0.001). There was also a significant difference between (urban 10(62.5%) and rural 5(26.3%) (χ2 = 4.644, sig (p) 0.0345) facilities with regards to designing and implementing a hand hygiene protocol that is in line with WHO recommendations. Concerning incinerators, 6 (62.5%) of urban centers and 14 (73.4%) of rural centers had insufficient incineration capacity in comparison to the generated wastes (**χ2** = 4.638, sig (p) 0.030).

## Discussion

The COVID-19 pandemic is currently testing countries’ health systems globally. However, it also offers a timely opportunity to stress the importance of water, sanitation, and hygiene (WASH) services in health care facilities [14]. In addition to being essential during pandemics such as the current Coronavirus, WASH services are integral for improving the quality of care, enhancing health systems, and ensuring the protection of patients as well as health care staff [23]. The goal of this study was to look into the availability, accessibility, and functionality of WASH facilities in temporary COVID-19 isolation and treatment centers of southern Ethiopia, as well as determine if there were any service differences between urban and rural sites.

It is no secret that performing effectively in any healthcare setting is something that requires the availability of appropriate facilities [24]. In the present study, 27 (77.1%) of the temporary treatment centers had daily water supply interruptions. Water from unimproved sources was also used by 7 (20%) of the treatment centers. Water quality has previously been reported to affect hospital efficiency and its deterioration is known to result in a drastic decrease in health services [25]. This is particularly true in COVID-19 treatment facilities [26].

The overriding need for a safe and improved water supply to prevail in the treatment centers was also the subject of an in-depth discussion. Observational findings offer further evidence for these sentiments. It was found that 20% of the treatment centers had water sources within 10 meters of contaminants. Poorly designed water distribution systems like this can enhance the growth of bacteria, resulting in the death of patients, particularly those with immune suppression [24, 27].

The availability of and accessibility to sanitation facilities varied for the different centers surveyed. 5 (14.28%) of the facilities did nothavetoilets separated for staff and patients, and only2 (3.57%) of the treatment centers had bathrooms that were handicapped accessible. As indicated by the WHO’s key guidelines on WASH in COVID-19 treatment centers, suspected or verified cases of COVID-19 have to be treated with separate flush lavatories that are not used by individuals who do not have COVID-19 [28]. In addition, sanitation observations made during the visits revealed that sanitary conditions were not up to the mark and needed to be addressed, particularly concerning the cleanliness of the toilet seats. However, the lack of funding in the study area was seen as contributing to the inability of the treatment centers to put in place such safety protocols.

Laundries are very important to prevent hospital-acquired infections [29]. All linen used by a person with confirmed, probable, or suspected COVID-19 infection must not be washed or dried by hand or by domestic washing machines [30]. In the present study, 94.7% of the laundries in the treatment facilities were not functional. The employer is responsible for ensuring a sufficient supply of required equipment to employees [31]. Failure to do so increases the risk of disease transmission dramatically. Similarly, 29 (82.85%) of the treatment centers had showers that were not functional or maintained properly (with 0.5% of chlorine after each patient). Cleaning and disinfection of high-touch areas and any objects that might have been in contact with body fluids should, where possible, be performed after every COVID-19 patient uses public washrooms [32]. However, the regular discontinuation in the supply of piped water makes it difficult if not impossible.

In low-income areas, providing access to water and soap will save lives until a Coronavirus vaccine becomes widely available [33]. Handwashing compliance in the clinical settings evaluated in this study was far from ideal. There were no usable handwashing stations in 9 (27%) of the treatment centers. This indicates that hand hygiene is not always taken as seriously as it should be. Governments must invest more in such services in light of the present rate of spread and death rates in many nations.

Waste generation amid COVID-19 has been a global environmental and public health crisis, particularly in developing and transition economies [34]. In addition to polluting the environment, the unsafe disposal of healthcare waste often contributes to the spread of infectious diseases [35]. Poor waste segregation, overfilling garbage bins, and inefficient garbage transportation and storage were all common occurrences in the current study. On-site and off-site waste disposal considerations should always be part of the design considerations when setting up such treatment centers [36].

Nearly all of the interview participants expressed a general shortage of PPE in the present study. Hospitals in low-income nations rely on the same supply chains to procure medical supplies as hospitals in wealthier countries, but they have far less negotiating power to secure resources [37]. Therefore, there is a need for sustained local and international action to ensure access to PPE for all health workers, not just those living in resource-rich countries.

Several reports on WASH services/infrastructure and practices in health care institutions in Africa and other developing countries have found a major urban-rural divide [38–40]. This was also evident in this study. According to the findings of the present study, there is a significant difference between urban and rural treatment centers concerning providing a functional, well-maintained, and improved latrine service. A study on Water, Sanitation, and Hygiene in Rural Health-Care Facilities across Kenya, Mozambique, Rwanda, Uganda, and Zambia reported similar results [41]. Rural communities have a low rate of improved latrine ownership, as evidenced by Ethiopian Demographic and Health Surveys (EDHS, 2016), which found that an estimated 56% of rural structures lacked improved latrines [42]. Given that these same buildings were turned into isolation and treatment centers, it appears plausible to presume that differences in latrine availability, functionality, and accessibility may be caused or exacerbated by the evaluated treatment centers’ geographic locations.

Several studies have shown that appropriate hand hygiene protocols lessen the risk of nosocomial infections, with estimates ranging from 23–53% [43]. People are more likely to wash their hands if they have access to clean water and soap. This reduces the risk of disease transmission [44]. In this study, there was a significant difference between rural and urban treatment centers when it came to implementing hand hygiene protocols that matched the WHO criteria. This is in line with studies done in North Western Ethiopia, India, and Nepal, which reported that one of the causes for low compliance of health institutions with recommended hand hygiene standards was a lack of supportive infrastructure resources in rural areas [45–47].

Poor healthcare waste management can lead to serious diseases for healthcare employees, waste collectors, patients, and the general public. Out-reach initiatives in developing countries frequently result in large amounts of waste being delivered to treatment centers in rural regions. And rural facilities that are within a reasonable distance of a legally recognized modern waste treatment or disposal facility are likely to be scarce [48]. In the present study, there was a considerable urban-rural disparity in the incineration capacities to manage generated healthcare waste. This is in line with research undertaken in Botswana, Nigeria, and Puerto Rico, which found that in rural regions, the treatment of healthcare waste using low-combustion incinerators and/or open burning and open disposal of incinerator ash is very widespread [48–50].

The present study has certain limitations. This is a particular sample in a single region and the findings are not likely to be representative of all treatment facilities in Ethiopia in their entirety. In Addition, this study did not assess actual handwashing practices among people, but it did examine the presence of hand-washing basins, soap, and posters or signs informing or reminding staff and patients about the need for handwashing. Nonetheless, the study’s advantages included a detailed and rigorous assessment of WASH facilities in temporary treatment centers, which encompassed concerns such as biological waste management and environmental hygiene in addition to water quality, sanitation, and hygiene.

## Conclusion

Efforts to improve WASH should offer priority to hygiene service interventions in treatment centers to minimize the risk of healthcare-acquired infections. The sustainable provision of hygiene services, such as water and handwashing soap, should also be given priority. This can be complemented by ensuring that information resources such as posters, leaflets, and signage on hand washing are available at key positions in the treatment centers. The poor toilets to the patient to caregiver ratios call for the provision of cheaper options for improved sanitation in these environments. Future research will benefit from a more systematic sampling plan that allows for the synthesis of bigger samples, to better represent temporary isolation and treatment centers across the country or in the Horn of Africa. Investigations should also be conducted to develop a comprehensive set of strategies to assist treatment centers in remote and rural locations to handle their wastes efficiently.

## Supporting information

S1 File Topic guide for key informant interviews. (DOCX)

## Abbreviations

COVID-19: Coronavirus Disease 2019
HCFs: Health care facilities
PPE: Personal Protective Equipment
SNNPR: Southern Nations, Nationalities and Peoples’ Region
WASH: water, sanitation, and hygiene
