# Supplementary material for: What it takes to save lives: An assessment of water, sanitation, and hygiene facilities in temporary COVID-19 isolation and treatment centers of Southern Ethiopia: A mixed-methods evaluation
Source: PLoS One. 2021 Aug 13;16(8):e0256086. doi: 10.1371/journal.pone.0256086 (PMC8362949; doi:10.1371/journal.pone.0256086)
Supplement: S1 File — (DOCX) [file pone.0256086.s001.docx]

**S1 File**

[Informed Consent Form for _________________________________]

This informed consent form is for health care workers in SNNPR who we are inviting to participate in a research, titled " What it takes to save lives: an Assessment of Water, Sanitation, and Hygiene Facilities in Temporary COVID-19 Isolation and Treatment Centers of Southern Ethiopia: A Mixed-Methods Evaluation``

**Aiggan Tamene**

**Wachemo University**

This Informed Consent Form has two parts:

- Information Sheet (to share information about the study with you)
- Certificate of Consent (for signatures if you choose to participate)
- You will be given a copy of the full Informed Consent Form

**Part I: Information Sheet**

My name is Aiggan Tamene and I am an Instructor at Wachemo University, College of Public Health. I would like to invite you to participate in a research topic entitled ‘What it takes to save lives: an Assessment of Water, Sanitation, and Hygiene Facilities in Temporary COVID-19 Isolation and Treatment Centers of Southern Ethiopia: A Mixed-Methods Evaluation’. Your participation is entirely voluntary, and you may choose not to participate. I am going to give you information and invite you to be part of this research. You do not have to decide today whether or not you will participate in the research. Before you decide, you can talk to anyone you feel comfortable with about the research. This consent form may contain words that you do not understand. Please ask me to stop as we go through the information and I will take time to explain. If you have questions later, you can ask them of me or of another researcher

# Purpose of the research

Quality water, sanitation, and hygiene facilities act as barricades to the transmission of COVID-19 in health care facilities. These facilities ought to also be available, accessible, and functional in temporary treatment centers. Despite numerous studies on health care facilities, however, there is limited information on the status of WASH facilities in such centers.

# Type of Research Intervention

This research will involve your participation in an interview that will take 15-20 minutes.

# Participant Selection

You are being invited to take part in this research because we feel that your experience as a health professional can contribute much to our understanding and knowledge of local health practices.

# Voluntary Participation

Your participation in this research is entirely voluntary. It is your choice whether to participate or not. You may change your mind later and stop participating even if you agreed earlier

# Procedures

During the interview, I or another interviewer will sit down with you in a comfortable place. If it is better for you, the interview can take place in your home. If you do not wish to answer any of the questions during the interview, you may say so and the interviewer will move on to the next question. No one else but the interviewer will be present unless you would like someone else to be there. The information recorded is confidential, and no one else except [name of person(s)] will access to the information documented during your interview.

# Risks

There is a risk that you may share some personal or confidential information by chance, or that you may feel uncomfortable talking about some of the topics. However, we do not wish for this to happen. You do not have to answer any question if you feel the question(s) are too personal or if talking about them makes you uncomfortable.

# Benefits

There will be no direct benefit to you, but your participation is likely to help us find out more about infection prevention practices in the southern region.

# Sharing the Results

Nothing that you tell us today will be shared with anybody outside the research team, and nothing will be attributed to you by name. The knowledge that we get from this research will be shared with you and your community before it is made widely available to the public. Each participant will receive a summary of the results. There will also be small meetings in the community and these will be announced. Following the meetings, we will publish the results so that other interested people may learn from the research.

# Right to Refuse or Withdraw

You do not have to take part in this research if you do not wish to do so, and choosing to participate will not affect you in any way.

# Who to Contact

If you have any questions or concerns about the study, please feel free to contact Aiggan Tamene at 09-13-99-45-30, apublic22@gmail.com. You may also contact Wachemo University Institutional Review Board for answers to questions about subject’s rights. Your willingness to assist with this project is deeply appreciated.

# Part II: Certificate of Consent

A researcher or the person going over the informed consent must sign each consent form. I have read the foregoing information, or it has been read to me. I have had the opportunity to ask questions about it and any questions I asked have been answered to my satisfaction. I consent voluntarily to be a participant in this study

Print Name of Participant__________________

Signature of Participant ___________________

Day/month/year ___________________________

- Statement by the researcher/person taking consent

I have accurately read out the information sheet to the potential participant, and to the best of my ability made sure that the participant understands that the following will be done

1. They will take part in the study

3. Their response will be anonymised and used for the study

I confirm that the participant was given an opportunity to ask questions about the study, and all the questions asked by the participant have been answered correctly and to the best of my ability. I confirm that the individual has not been coerced into giving consent, and the consent has been given freely and voluntarily.

A copy of this ICF has been provided to the participant.

Print Name of Researcher/person taking the consent________________________

Signature of Researcher /person taking the consent__________________________

Date _______________________

**Additional file 1:** Topic Guide

Assessment of Water, Sanitation, and Hygiene (WASH) Facilities in Temporary COVID-19 Isolation and Quarantine Centers of Southern Ethiopia

Note: This topic guide is indicative

**Introduction**

- Thank you for participating
- Self-introduction
- Introduce the study
- Talk about key points:
- duration of the interview
- interview like a discussion, but will cover key topics
- no correct or incorrect answers
- voluntary participation, rights to withdraw
- Confidentiality and anonymity, how to disclose findings
- Questions?
- Ready to continue? Sign a form of consent

**START RECORDING**

**Topic 1**- Respondent profile

- To begin with, tell me about yourself and your work here
- Tell me about your specific tasks in the hospital
- How long have you worked here?

**Topic 2-** Background/general situation in treatment center: How do you judge the water, sanitation, and hygiene in your center? Please explain it so I may understand your day to day experiences.

**Topic 3**- During the months since the start of the pandemic, how many times was the water supply from this source interrupted for more than two hours at a time?

**Topic 4**- What are the barriers that limit the WASH facilities in your treatment center? Please explain from your perspective

**Topic 5-** What are the sources of these problems? Give me your opinion of why you think the problem persists

**Topic 6**- Looking to the future…Tell me about how you think the WASH problems may be solved

**Closing**

- Anything to add? Anything that you feel is missing?
- Any questions?
- Copy of interview transcript?
- Summary of results from the study?

**Thank you very much!**

# Section II: Observational Checklist

**ASSESSMENTOF HEALTH CARE FACILITIES FORCOVID-19 RESPONSE**

**CHECKLIST – WASH COMPONENT**

| Date of assessment: ______________ | Name/Organization/contact of team   1. _______________________________________ 2. _______________________________________ 3. _______________________________________ 4. _______________________________________ |
| --- | --- |

| **General Information** | |
| --- | --- |
| Type of the facilities:___________________  Name: _______________________________  District/Location/Zone: __________________  Bed Capacity: ________________________  Expected number of staff: _______________  Occupancy Rates: _________________% | Water demand (# patients and staff X 150l/day): __________  Expected grey water (80% of water demand):_____________  Expected black water (20% of water demand):_____________  Expected fecal matter in black water (0.5l/day X #patients and staff):________________________________ |

| **WATER SUPPLY** | |
| --- | --- |
| **WATER QUANTITY**  Insufficient water quantity for all the daily needs in the health facility.  Daily interruptions in water supply at the health facility.  Insufficient water storage (less than 24 hours backup supply). | **Comments** |
| **WATER QUALITY**  Water is from an unimproved source or sources of contamination (latrines, waste, pollution, etc.) within 10m / 33ft of the water source.  Water is un-chlorinated, insufficiently chlorinated (no chlorine smell or taste in the water at the tap) or is turbid (cloudy).  Broken water pipes or uncovered or unsanitary water reservoirs.  Drinking water for staff, visitors and patients is not safe and/or in inadequate quantity. | **Comments** |
| **WATER DISTRIBUTION**   Some units do not have dedicated water points.   All the water lines to WASH facilities does not have measures to prevent contamination of the system.  At random open water taps, the water is flowing without enough pressure. | **Comments** |

| **EXCRETA DISPOSAL** | | |
| --- | --- | --- |
| **EXCRETA DISPOSAL**  Facility does not have toilets separated for staff, patients, and visitors.  Some units do not have dedicated latrines.   Existing toilets are not separated by sex and for people with reduced mobility.   Latrines are not maintained properly.   Latrines are not regularly (every 2-3 hrs) disinfected.  Not all latrines have dedicated handwashing stations with water and soap or chlorinated water.   Some pits and/or septic tanks are full. | **Comments** | |
| **LIQUID WASTE** | | |
| **DRAINAGE**   Pools of standing water observed at water points.   Potentially infectious wastewater from bathing, cleaning or laundering activities visible in the health facility environment.   Stormwater drains or canals blocked, non-existent, or non-functional. | **Comments** | |
| **SHOWERS AND LAUNDRY** | | |
| **SHOWERS AND LAUNDRY**   Some units do not have dedicated showers.   Showers are not functional or maintained properly (with 0.5% of chlorine, after each patients).  Laundry area is not functional.   Some soak-away pits are full. | **Comments** | |
| **HANDWASHING STATIONS** | | |
| **HANDWASHING**   Absence of soap or chlorinated water at any handwashing locations.   Absence of posters reminding users of correct handwashing procedures.   Absence of functional hand washing points in any location where healthcare is delivered (wards, consulting rooms, delivery rooms, operating theatres, etc.) or service areas (kitchen, laundry, toilets, waste zone, mortuary, etc.).  Absence of hygiene promotion and handwashing supplies and stock-piling.   Patients and caring people not informed of essential hygiene behaviors necessary for limiting disease transmission within 30 minutes of arrival.  Hand hygiene protocol is properly designed and applied regularly. | **Comments** | |
| **SOLID WASTE MANAGEMENT** | | |
| **SOLID WASTE MANAGEMENT**   Insufficient, inadequate or overflowing waste disposal containers.   No sources of separation of hazardous wastes (e.g. infectious, non-infectious, sharps).  Medical wastes (needles, dressings, etc.) observed in health facility grounds or public spaces or medical waste disposal area unfenced?  Incinerator capacity is not sufficient compared to the waste generated by the HCF.  Inexistent of third party company to carry the waste for incineration (this is CPH convention that all HCF to have a third party to manage the hospital waste). | | **Comments** |
| **INFECTIONPREVENTION AND CONTROL (IPC)** | | |
| **INFECTION PREVENTION AND CONTROL (IPC)**  Inadequate disinfection of beds, floors, walls, equipment, surfaces, or inadequate disposal of faeces and hygiene items from infectious patients.   Lack of disinfection of hands (with hand sanitizer or 0.5% chlorine solution in the ward and 0.05% outside).   Lack of cleaning equipment (buckets, mops, etc.) or disinfectant solutions.     Lack of personal protection equipment (gloves, overalls, masks, etc.) for staff.  Insufficient staff to demonstrate cleaning and disinfection.  Lack or insufficient stock of IPC-related supplies.  Lack of record keeping of cleaning activities undertaken by the cleaners each day. | | **Comments** |

| **RECOMMENDATION AND COMMENTS** |
| --- |
|  |

**1. የመረጃቅጽ**

ሰላምታ፡-ጤናይስጥልኝ!! እኔ**__________________**እባላለሁ፡፡ይህጥናትየሚካሄደውአቶ ኤገን ታመነ በተባሉትየዋቸሞዩኒቨርሲቲህክምናናጤናሳይንስኮሌጅመምህርሲሆንበዩኒቨርሲቲውሙሉፈቃድታግዘውየበክልሉበተመረጡህለክምናማእከላትላይየውሃ፣መጸዳጃ፤ ቆሸሸ ስርአት ያለበትደረጃለመለየትናከአገልግሎቱጋርበተያያዘያሉተያያዥሁነቶችንለመለየትነው፡፡ይህንመጠይቅለመሙላትከ30 እስከ 40 ደቂቃየሚወስድሲሆንበዚህቃለመጠይቅፈቃደኛካልሆኑያለመሳተፍመብትዎ የተጠበቀ ነው፡፡እርስዎበዚህጥናትተሳታፊበመሆንዎበቀጥታሊያገኙየሚችሉትነገርላይኖርይችሊል፤ነገርግንየእርስዎተሳትፎበአገልግሎቱአጠቃቀምናምክንያትዙሪያያሉችግሮችወይምክፍተቶችለማሳየትእናትክክለኛየመፍትሔአቅጣጫለመጠቆምእጅግአስፈላጊነው፡፡ጥናቱውጤታማሊሆንየሚችለውእርሶበሚሰጡትትክክለኛመልስላይየተመረኮዘበመሆኑ፤ጥያቄዎቹንበጥንቃቄእንዲመልሱልንፍቃደኝነትዎንበትህትናእንጠይቃለን፡፡በተጨማሪምየሚሰጡትመረጃከተባለለትጉዳይውጪእንደማይውልእናሚስጥራዊነቱየተጠበቀእንደሚሆንአረጋግጣለሁ፡፡በቃለመጠይቅወቅትለእርስዎግልጽያልሆነነገርካለመጠየቅይችላሉ፡፡ለመመለስፈቃደኛያልሆኑበትጥያቄካለምማለፍይችላሉ፡፡በየትኛዉምምክንያትበመጠይቁመሃልማቋረጥቢፈልጉጥያቄዉንየማቋረጥመብትአለዎት፡፡

ተጨማሪጥያቄካለዎትናእንዲሁምበቀጣይየተሰበሰበውመረጃውጤትለማወቅከፈለጉከዚህበታችበተጠቀሰውየዋናውየጥናቱባለቤትስልክቁጥርናአድራሻተጠቅመውማግኘትይችላሉ፡፡

እርሶምበዚህጥናትበመሳተፎከልብአመሰግናለሁ!!!!!!

ኤገን ታመነ

ስልክቁጥር +251913994530

አድራሻ:-[apublic22@gmail.com](mailto:apublic22@gmail.com)

**2. የስምምነትቅጽ**

ከሊይየተፃፈውንየመረጃቅፅአንብቤየጥናቱንአላማናጥቅምበግልጽተረዴቻለሁ፡፡በዚህምመሰረት

ያለጥናትቡድኑአባላትተፅእኖበሙሉ ፈቃደኝነትበዚሁጥናትበመሳተፍበወረዳውያለውየተማላየድህረወሊድአገልግሎትተጠቃሚነትናተያያዥጉዳዮችዙሪያያለውሁኔታለመለየትበሚደረገውጥናትላይየሚጠበቅብኝንአስተዋፅኦለማበርከትመወሰኔንበፊርማዬአረጋግጣለሁ፡፡

የተሳታፊውቁጥር ________**_______**ፊርማ**______________**ቀን___________

የመረጃሰብሳቢሥም ________________________ ፊርማ ________________

መረጃየተሰበሰበበትቀን____________ የተጀመረበትሠዓት ______ ያለቀበትሰዓት _______

**ክፍል ሁለት፡ መሪ ጋይድ**

- ለተሳትፎ እናመሰግናለን
- ራስን-ማስትዋውቅ
- ጥናቱን ማስተዋውቅ
- መሰረታዊ ሀሳቦቸ
- የኢንተርቪው ደቂቃ
- መልስ ልክም ሊሆን ላይሆንም ይቸላል
- በበጎ ፍቃድ ታሳትፎ ሚጠና ጥናት
- ምሰጢራዊነት
- ጥያቄ

**መቅዳት መጀመር**

ርእስ 1- የተሳታፊ ፕሮፋይል

- ለመጀመር ስለ ራሳሶት ንገሩን
- በዚህ ሆስፒታል ውስጥ ስራዎት ምንድነው
- በዚህ ቦታ ስንት ጊዜ ሰሩ

ርእስ 2- በዚህ ህክምና ማከል ውስጥ ያለውን የውሃ፣መጸዳጃ፤ ቆሸሸ ስርአት ምን ይመስላል. በሚመቸሆት መልኩ አስርዱን፣ ልምዶትንም ያካፍሉን

ርእስ 3- ከኮሮና ቫይረስ በሸታ በሁላ ፣ ውሃ አቅርቦት ምን ይመስላል፣ መቆራርጥ አለው

ርእስ 4- የውሃ፣መጸዳጃ፤ ቆሸሸ ስርአት ማነቆዎቸ ምንድናቸው፣ ከናንተ አመለካከት አን*ፃር*

ርእስ 5- የቸግሮቸ መንስኤ ምንድናቸው፣ አስተያቶን ይንገሩ

ርእስ 6- ለወደፊት የውሃ፣መጸዳጃ፤ ቆሸሸ ዙርያ ምን ይታያቸሁዋል

**መዝጊያ**

- ያላለቀ ነገረ ካል
- ጥያቄ ካሎት
- የትራንስክሪፕቱ ኮፒ
- የውጤት ኮፒ

እናመስግናለን

**ክፍል 3- እይታ ሊስት**

| ***ውሃ*** | |
| --- | --- |
| ***የውሃ አቅርቦት***  በቀን ውስጥ ለሁሉም ግልጋሎት በ*ቂ የሚሆን የውሃ አቅርቦት የለም*   *በዚህ ህክምና ማከል ውስጥ በየቀኑ ውሃ ትጠፋለቸ*  *በዚህ ህክምና ማከል ውስጥ* በ*ቂ የሚሆን የውሃ ክምቸት የለም* | **ተጨማሪ ሃሳብ** |
| ***የውሃ ጥራት***  የሚቀርብላቸሁ ውሃ ከበካይ ነገሮቸ (ላትሪን፤ቆ*ቫቫ*፣ብክለት) በ10 ሜትር ርቀት ውስጥ ነው  ውሃው ክሎሪን ያላገ*ኛው፣ ወይንም በበቂ ሁኔታ* ያላገ*ኛው ነው (የ*ክሎሪን *ሸታ ወይንም ጣም የሌለው)*  የተሰበሩ ወይንም ያልት*ሸፈኑ ቱቦዎቸ፤ ቆቫቫ ውሃ ማጠራቀምያዎቸ አሉ*  *በቂ የመጠጥ ውሃ ለስታፍ፣ ለታካሚ፣ ለጠያቂ አለ* | **ተጨማሪ ሃሳብ** |
| ***የውሃ ስርጪት***  *አንዳንድ ዩኒቲች (ቦታዎቸ) የውሃ አቅርቦት የላችውም*  የ*ውሃ ቱቦዎቸን* ከበካይ ነገሮቸ (ላትሪን፤ቆ*ቫቫ*፣ብክለት) *ማራቅ ተችሎአል*  *አንዳንድ ቡዋንቡዋዎቸ በቂ የፍሰት አቅም የላቸውም* | **ተጨማሪ ሃሳብ** |

| **መ*ፃዳጃ አገግሎት*** | |
| --- | --- |
| *በዚህ ህክምና ማከል ውስጥ* መ*ፃዳጃ አገግሎት ለሰራተኛ፣ ለታማሚ፣*  *ለአስታማሚ ተከፋፍሎ አለ*  *አንዳንድ ህክምና ማከሎቸ የራሳቸው* መ*ፃዳጃ የላቸውም*   መ*ፃዳጃ ያላቸውም ለውንድ ለሴት ተብለው አልተከፈሉም*   መ*ፃዳጃ ቤቶቸሁ በየ 2-3 ሰአቱ አየጥታጠቡም*  *ሁሉም* መ*ፃዳጃ ቤቶቸ የራሳቸው መታጠብያ*  *አልተስራላቸውም*  *አንዳንድ ሰገራ ማጠራቀምያ ገንዳዎቸ ሞልተዋል* | **ተጨማሪ ሃሳብ** |

| **ፍሳ*ሽ*** | |
| --- | --- |
| **ፍሳ*ሽ* ማስወገድ**  *የተጠራቀመ ውሀ በውሀ መቅጃ ና ማጠራቀምያ ቦታዎቸ ላይ ይታያል*  *በሽ* ታን የማምጣት *አቅም ያለው ውሀ (ከመታጠብያ ክፍል፣ ከላውንዳሪ፣ ከመፃዳጃ ክፍሎቸ ፈሶ የህክምና ማከሉ ውስጥ ይታያል*  *የጎርፍ ማስገጃ ትቦዎቸ ተደፍነው ወይንም ከጥቅም ው*ጨ ሆነው ይታያሉ | **ተጨማሪ ሃሳብ** |

| ***የመታጠብያ ክፍል እና ከላውንዳሪ*** | |
| --- | --- |
| *አንዳንድ ህክምና ክፍሎቸ የራሳቸው የመታጠብያ ክፍል አልተስራላቸውም*   *አንዳንድ* *የመታጠብያ ክፍሎቸ በ አግባቡ አይተጠቡም*  (0.5 % ክሎሪን፣ ታካሚ ገላውን ከታጠበ ቦሀላ)  *በዚህ ህክምና ማከል ውስጥ ላውንዳሪው ስራ ላይ ነው*  *አንዳንድ* *የልብስ መዘፍዘያዎቸ ሞልትው ይታያሉ* | **ተጨማሪ ሃሳብ** |

| ***የእጅ መተጠብያ*** | |
| --- | --- |
| *ሳሙና ወይንም ክሎሪኔትድ ውሃ በእጅ መተጠብያ ቦታዎቸ አለመኖር*  *የእጅ መተጠብያ ስርአትን አመላካቸ ፖስተሮቸ የሉም*  *ጥቕም ላይ ያሉ (አገልግሎት የሚሰጡ ) የእጅ መተጠብያ ቦታዎቸ በሁሉም የጤና መስ*ጨ ቦታዎቸ *አሉ*  *የእጅ መተጠብያ ስርአትን አመላካቸ ፖስተሮቸ በስቶር ውስጥ ተጠራቅመው ይገኛሉ*  *ለታካሚ፣ አስታማሚ፣ ለጠያቂ የእጅ መተጠብያ ስርአትን ይነገራቸዋል*  *የእጅ መተጠብያ ፕሮቶኮል ዲዛይንድ ሆኖ በስራ ላይ ውሉአል* | **ተጨማሪ ሃሳብ** |

| ***ደረቅ ቆሻሻ አወጋገድ*** | |
| --- | --- |
|  *በቂ ያልሆነ፣ የሚያፈስ ደረቅ ቆሻሻ መጣያ ገንዳዎቸ አሉ*   *የአደገኛ ቆሻሻ በሆስፒታሉ ግቢ ውሰጥ ይታያል (በመከለያም ተገድቦ አልተቀመጠም)*   *የሆስፒታል ቆሻሻ ማቃጠያ ከሚመረተው ቆሻሻ ጋር ሲታይ በቂ አይደለም*   *በሶስተኛ ወገን/ድርጅት ቆሻሻ የሚያቃጥል ወገን አለ* | **ተጨማሪ ሃሳብ** |

| ***ኢንፌክሽን መከላከል እና መቆጣጠር*** | |
| --- | --- |
|  *የአልጋ፣ መሬት፣ ግድግዳ፣ እቃ አልአግባብ ኢንፌክሽን መከላከል ስራዎቸ እንዲሁም አልአግባብ የሰገራ አወጋግድ እና ንፃህና ያልተጠበቀ አካባቢ ከተላላፊ በሽታ ህመምተኛዎቸ ይታያል*   *የ እጅ ዲስኢንፌክሽን ቦታዎቸ በየዋርዱ ይታያሉ (የእጅ ሳኒታይዘር ወይንም 0.5 ፕረስነተ ክሎሪን አለ)*   *የማጠያ እና የንፃህና እቃዎቸ (መዘፍዘፍያ፣ መጥደግያ፣ዲስኢንፌክታንት ሶሉሽን )በጤና ማከሉ የሉም*   *የራሥ መከላክያ መሳርያዎቸ (ማስክ፣ኦቨረኦል፣ግለቭ) ለስታፍ ይገኛሉ*   *ኢንፌክሽን መከላከል እና መቆጣጠር ሰራዎቸን ለማስትማር በቂ የስው ሃይል የለም*   *ኢንፌክሽን መከላከል እና መቆጣጠር ሰራዎቸን ልማገዝ የሚረዱ እቃዎቸ በበቂ ሁኔታ አሉ*   *የተስሩ ኢንፌክሽን መከላከል እና መቆጣጠር ስራዎቸን በአግባቡ ዶክምነት የማርግ ልምድ አለ* | **ተጨማሪ ሃሳብ** |

| **ተጨማሪ ሃሳብ እና አስትያየት** |
| --- |
|  |
